# Uncontrolled Semantic Adaptation in Clinical Evaluation of Large Language Models

**DOI:** 10.1016/j.mcpdig.2025.100309

**Published:** 2025-12-06

**Authors:** Alfredo Di Giovanni

**Affiliations:** Ophthalmologist and Artificial Intelligence Researcher, Ophthalmology Service, ASL Napoli 2 Nord, Naples, Italy

To the Editor:

Large language models (LLMs) are increasingly evaluated for clinical interpretation and decision-support tasks. Several studies have compared their diagnostic performance, often assuming that identical prompts and models yield comparable results across users. However, this assumption may not hold true.

Repeated use of an LLM by a clinician within a specific diagnostic field can induce a progressive semantic adaptation: the model implicitly aligns with the linguistic and conceptual patterns that dominate the user’s previous interactions. Consequently, even when identical queries and identical model architectures are used, performance may differ depending on whether the access occurs through an account that has previously addressed similar problems or through a completely new, unexposed account.

This divergence does not represent true learning or fine-tuning of the model, but rather an emergent contextual alignment driven by previous conversation history. In clinical testing, such uncontrolled adaptation may inflate apparent accuracy in users with rich domain-specific interaction histories, while underestimating performance in clean conditions.

As comparative evaluations of LLMs for medical use proliferate, this hidden semantic adaptation bias threatens reproducibility and fairness. Reported differences between models—or between user groups—may reflect unequal exposure rather than intrinsic diagnostic capability.

We recommend that all future LLM performance studies explicitly control for user history, standardize interaction conditions, or employ freshly initialized accounts. Without these safeguards, observed disparities may conflate contextual bias with genuine clinical intelligence.

## Potential Competing Interests

The author report no competing interests.

## Declaration of Generative AI and AI-Assisted Technologies in the Writing Process

During the preparation of this work, the author used ChatGPT (OpenAI, GPT-5 model) in order to assist in language editing and refinement of the English text. After using this tool, the author reviewed and edited the content as needed and takes full responsibility for the content of the publication.

